# School masking and COVID-19 community transmission: a synthetic control study

**DOI:** 10.1093/haschl/qxaf233

**Published:** 2025-12-23

**Authors:** Xianqun Luan, Brian T Fisher, Susan E Coffin, David Rubin, Meredith Matone, Jing Huang

**Affiliations:** PolicyLab, Children's Hospital of Philadelphia, Philadelphia, PA 19104, United States; Division of Infectious Disease, Children's Hospital of Philadelphia, Philadelphia, PA 19104, United States; Division of Infectious Disease, Children's Hospital of Philadelphia, Philadelphia, PA 19104, United States; Office of the President, University of California, Oakland, CA 94607, United States; PolicyLab, Children's Hospital of Philadelphia, Philadelphia, PA 19104, United States; Department of Biostatistics, Epidemiology, and Informatics, Perelman School of Medicine, University of Pennsylvania, Philadelphia, PA 19104, United States

**Keywords:** synthetic control, K-12 schools, pandemic preparedness

## Abstract

**Introduction:**

K-12 schools are not only educational settings but also hubs of social interaction, making them potential drivers of disease transmission within households and communities. While many existing studies have assessed school masking in relation to in-school transmission, the broader community impact of mandatory school masking policies on SARS-CoV-2 infection rates remains poorly understood.

**Methods:**

We conducted a retrospective quasi-experimental study using the synthetic control method to evaluate the association between masking policies and community infection rates during the fall 2021 US school reopening period, when most schools returned to in-person learning but masking policies varied substantially. Analyses accounted for community characteristics prior to reopening and baseline infection rates.

**Results:**

Counties with mandatory school masking experienced significantly lower SARS-CoV-2 infection rates than those without mandates. In the first 9 weeks after reopening, mandatory masking was associated with 820 fewer cases per 100 000 people (95% CI: 444-1185), corresponding to a relative cumulative reduction of 9.4% (95% CI: 7.3%-11.8%). The strength of this association varied by baseline infection rates, population density, and mobility patterns.

**Conclusion:**

Mandatory school masking policies were linked to meaningful reductions in community SARS-CoV-2 transmission and underscore their value as a public health intervention during pandemic surges.

## Introduction

Kindergarten through 12th grade (K-12) schools are unique environments with extensive human interaction, where children closely interact with peers and then return home to engage with their families. This dynamic creates a potential pathway for infectious diseases, like SARS-CoV-2, to spread not only within schools but also into the surrounding community. Masking policies in schools were implemented as a critical non-pharmaceutical intervention during the COVID-19 pandemic, aiming to reduce transmission in these high-contact settings. While numerous studies have examined the effects of school masking policies on secondary transmission within school environments and the impact of community masking policy on community SARS-CoV-2 incidence rates, the relationship between mandatory masking policies in public schools and broader community transmission has been less thoroughly investigated.^1-4^

In this study, we aim to examine whether mandatory school masking policies, compared to non-mandatory policies, influenced community SARS-CoV-2 incidence rates during the Fall of 2021, a period when most schools had returned to in-person learning, and masking policies varied significantly across different regions of the United States.^5^ We also explore how community characteristics, such as population density, socioeconomic factors, and local SARS-CoV-2 incidence rates, may have shaped the effectiveness of these policies.

## Methods

### Study design

This retrospective study focused on US counties with a single public school district to reduce within-county variation in school policies and facilitate clearer attribution of outcomes. We adopted county selection criteria developed in a prior study assessing impact of instructional modality on disease transmission,^6^ prioritizing counties with adequate population sizes to ensure stable estimates of community incidence rates. Among the 229 counties that met these criteria, 166 counties were selected to ensure sufficient variation in school masking policies within states or in nearby states. This heterogeneity in exposure was essential for evaluating the effects of school masking policies while accounting for regional similarities in demographics, policy environments, and COVID-19 transmission dynamics. Specifically, the selected counties were located in states where both mandatory and non-mandatory school masking policies were present or in states bordering such states at the start of the Fall 2021 semester. Within this subset, 89 counties implemented mandatory school masking policies, while 77 counties implemented non-mandatory masking policies. For each county, time zero was defined as the start date of the Fall 2021 academic semester. County-level data for the analyses were collected over a 15-week period, spanning 6 weeks before and 9 weeks after time zero (Fall semester 2021 school opening date).

### Intervention

The intervention was the county-level K-12 public school masking policy, categorized as mandatory vs non-mandatory masking. Hereafter, we refer to schools with mandatory school masking policy as *treated* and schools with non-mandatory masking policies as c*ontrol*. Masking policy data were extracted from official documents published on school and district websites and classified as follows: (1) mandatory masking (*treated*)—school districts that required masking at the start of the Fall 2021 semester and (2) non-mandatory masking (*control)*—schools districts where masking was optional, recommended, or applied in limited settings (eg, hallways, grade-specific recommendations) at the start of the Fall 2021 semester. To ensure the accuracy of masking policy classification, a blinded independent data collection and validation process was conducted for a random 20% sample of school districts, yielding 92% agreement between initial and validation coding.

### Outcome

The main outcome was the weekly SARS-CoV-2 incidence rate per 100 000 residents at the county level. Daily incidence data were sourced from USAFacts, and population size estimates were obtained from the US Census Bureau.^7,8^ Both confirmed and probable cases were included following CDC guidelines.

### Covariates

County-level covariates were included to account for potential confounding factors, encompassing demographics, health-related variables, mobility data, and vaccination rates. Key data sources included demographics derived from the American Community Survey Data,^8^ including population density, urban population rate, senior population (≥64 years), young population (≤18 years), minority, college education, high school diploma, poverty rate, and supplemental security income; health-related factors acquired from the County Health Rankings & Roadmaps,^9^ including any chronic condition, obesity, diabetes, smoking, and excessive drinking; social vulnerability obtained from the CDC/Social Vulnerability Index^10^; and mobility data obtained from Google COVID-19 Community Mobility Reports,^11^ with weekly mobility calculated as a 7-day average of daily mobility metrics across various categories, such as visits to retail and recreation, grocery and pharmacy, workplaces, and residential areas and SARS-CoV-2 vaccination rates data obtained from CDC.^12^ These covariates were selected a priori based on existing evidence of factors influencing county-level SARS-CoV-2 incidence rates.^13-15^

Given a large number of covariates, we conducted a principal component analysis (PCA) of county-level covariates to achieve a reduced-rank presentation.^16^ The top 5 principal components (PCs), which collectively accounted for over 77% of the total variance of the covariates, were selected. This approach was used to summarize correlated covariates into a smaller set of uncorrelated components, improving interpretability and reducing noise from multicollinearity in subsequent analyses. The variable loadings and interpretation of the top 5 PCs are provided in Figure S1.

### Synthetic control group construction

We evaluated the effect of school masking policies on county-level SARS-CoV-2 incidence rate using the synthetic control method (SCM).^17-20^ SCM is particularly well-suited to comparative case studies evaluating the impact of a policy intervention implemented at an aggregate level (here, counties), with a limited number of treated units and a well-defined intervention timing. It explicitly balances both baseline covariates and pre-intervention outcome trajectories, which are strong predictors of future incidence in epidemics. While multivariable regression and propensity score methods can adjust for observed covariates, and propensity score models can include summaries of pre-intervention outcomes, they do not guarantee close alignment of entire pre-policy trajectories at the unit level. By constructing a weighted synthetic counterpart for each treated county that reproduces its pre-policy path, SCM reduces bias from unmeasured, time-invariant confounding and provides transparent fit diagnostics.

For each treated county, a synthetic control was constructed as a weighted combination of all eligible control counties from a donor pool specifically built for the treated county. These control county pools, primarily selected from the same state as the treated county, aimed to provide at least 5 control counties when available. If fewer than 5 control counties were available within the same state, the donor pool was expanded to include control counties from geographically adjacent states to account for regional similarities in population characteristics, policy environments, and SARS-CoV-2 transmission dynamics. The resulting donor pool sizes ranged from 7 to 32 counties per treated county.

County weights for the synthetic controls were calculated using pre-intervention SARS-CoV-2 outcomes and the top 5 PCs of county-level covariates to minimize differences between treated counties and their corresponding synthetic controls. The quality of the constructed synthetic controls was assessed by the absolute standardized mean difference (ASMD), with an ASMD threshold of 0.5 or less considered indicative of a well-matched synthetic control.^21^ Additional details on the construction of the synthetic controls are provided in the Supplementary Materials. The resulting synthetic controls made up the synthetic control group.

### Statistical analysis

The impact of the intervention was estimated as the differences and ratios in weekly SARS-CoV-2 incidence rate between treated counties and their synthetic counterparts during the 9-week post-intervention period. Cumulative incidence rate over this 9-week period was also calculated. The 95% CIs were derived using the bootstrap method with 1000 resamples.

To assess variation in intervention effects, we conducted stratified analyses across several subgroups. Treated counties were categorized into high and low groups for each of the following pre-intervention metrics: cumulative SARS-CoV-2 incidence rates, population density, and community mobility. Median values of each metric within the treated counties during the pre-intervention period were used as cutoffs to define high and low categories. Intervention effects were then summarized for each category and compared across the strata. A placebo check was conducted by applying the same synthetic control procedure to control counties as if they had implemented the policy to assess whether the estimated effects were observed by chance. Sensitivity analyses were conducted to assess the robustness of the synthetic control construction. Details on the placebo check and sensitivity analyses are provided in the Supplementary Materials. Analyses were conducted in R (version 4.4.0), using the Synth package.

## Results

**Figure 1.**
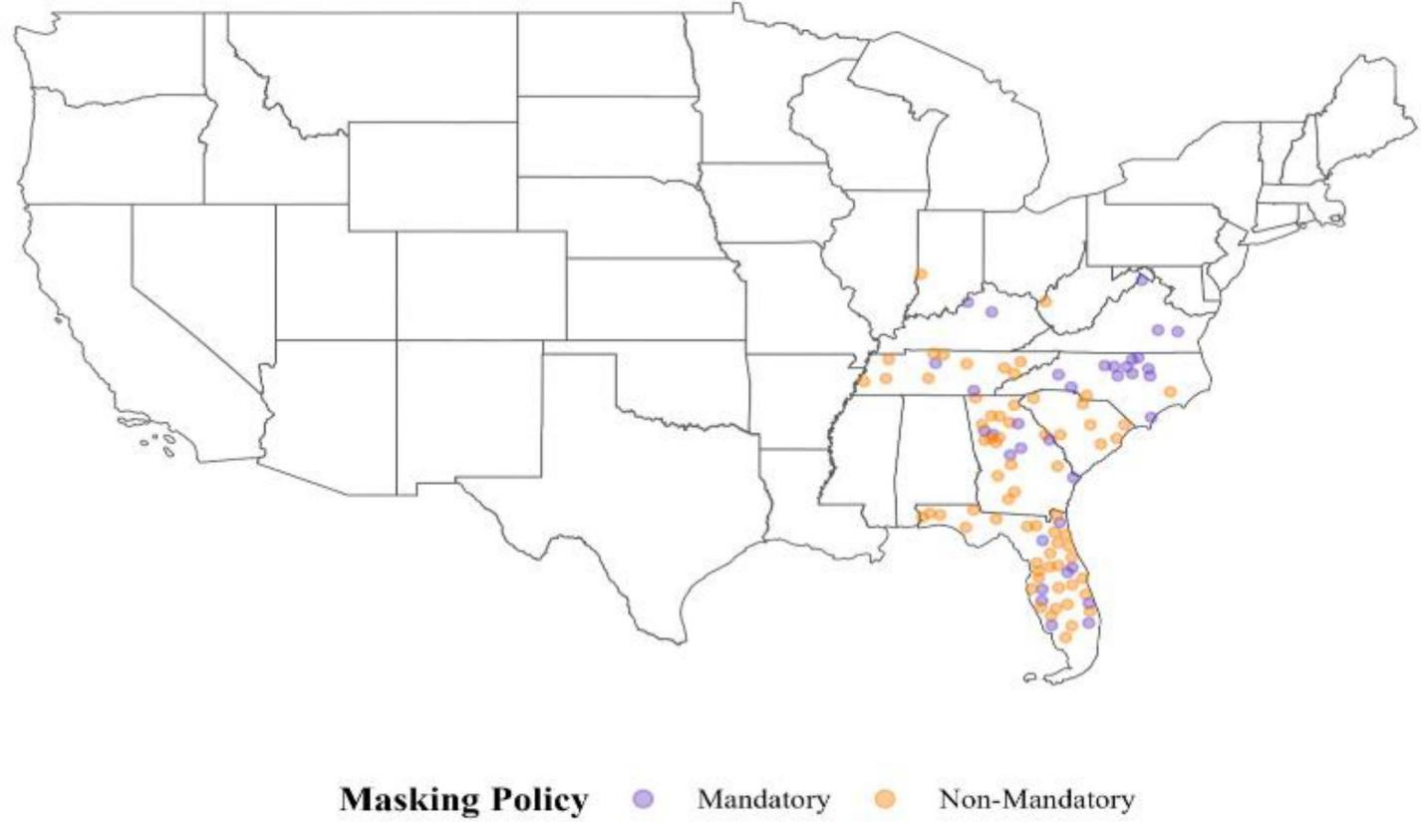
Geographic locations of counties included in the final analysis and their school masking policies at the start of the Fall 2021 semester. This includes 35 treated counties and the 69 control counties used to construct their synthetic controls.

The final analysis included 35 treated counties along with their corresponding synthetic controls, constructed from 69 control counties. These treated counties and their synthetic counterparts achieved an ASMD of ≤ 0.5 for pre-intervention covariates and outcomes. A flowchart outlining county selection and synthetic control construction is provided in Figure S2. Variation in school masking policies was most pronounced in the southern regions of the United States, leading to an overrepresentation of treated and control counties from the South Atlantic and East South-Central regions in the final study sample (Figure 1). Figure S3 shows the geographic locations and corresponding weights of control counties used to construct the synthetic control for each treated county, while Table S1 summarizes the total weights assigned to each control county, indicating their relative contribution to the synthetic control group. The analytic sample was reduced from 89 to 35 treated counties after applying pre-intervention balance restrictions. Excluded and retained counties were broadly similar, with only modest differences in vaccination coverage and urbanicity (Table S2).

Table 1 shows summary statistics of baseline demographics and SARS-CoV-2 related measures during the pre-intervention period for the 35 treated counties, the 69 control counties used to construct the synthetic controls, and the 35 synthetic controls. Compared to control counties, treated counties had younger populations, were more urban, and had higher population density, higher educational attainment, and higher SARS-CoV-2 vaccination coverage. They also had lower proportions of adults aged 65 years or older, lower prevalence of chronic conditions and smoking, lower public insurance coverage, and reduced mobility. The percentage of the population infected with SARS-CoV-2 prior to the start of the Fall 2021 semester was slightly lower in treated counties (10.9% vs 11.1%). Weekly SARS-CoV-2 incidence rates were also lower in treated counties during the 6 weeks preceding the semester's start, with rates of 337.0 vs 418.0 per 100 000 population in the week immediately before schools opened. These baseline differences were substantially reduced after constructing the synthetic controls. Comparisons of pre-intervention outcomes and county-level covariates between treated counties and their synthetic controls indicate a close balance between the groups, as reflected by small mean differences.

**Table 1.** Summary of pre-intervention outcomes and county-level covariates, reported as mean with standard deviation in parentheses, during the 6 weeks before the Fall 2021 semester, comparing counties with mandatory vs non-mandatory school masking policies and mandatory-masking counties vs their synthetic controls.

	Treated counties	Control counties	Synthetic controls	Treated vs control counties^c^	Treated vs synthetic controls^c^
**# Counties**	35	69	35		
**Weekly SARS-CoV-2 case rate per 100 K before the Fall 2021 semester**					
6th week before school opening (week −5)	38.3 (29.2)	41.5 (35.4)	37.9 (27.6)	−3.2	0.4
5th week before school opening (week −4)	57.1 (43.0)	87.2 (147.7)	56.2 (43.8)	−30.1	0.9
4th week before school opening (week −3)	200.0 (249.1)	264.3 (278.7)	192.9 (248.7)	−64.3	7.1
3rd week before school opening (week −2)	228.6 (215.8)	289.2 (252.4)	228.7 (213.7)	−60.6	−0.1
2nd week before school opening (week −1)	276.6 (180.5)	333.2 (234.2)	279.4 (180.6)	−56.6	−2.8
1st week before school opening (week 0)	337.0 (180.1)	418.0 (258.0)	340.4 (175.1)	−81	−3.4
**Population percentage of reported SARS-CoV-2 infection (%)**	10.9 (1.9)	11.1 (2.6)	11.5 (1.8)	−0.2	−0.6
**Population percentage of COVID-19 vaccination** ^a^	38.6 (14.0)	35.5 (13.0)	37.0 (10.3)	3.1	1.6
**Population demographics**					
Population Density in log scale	6.3 (0.8)	5.5 (0.8)	6.0 (0.7)	0.8	0.3
% Urban population	82.4 (18.5)	71.0 (18.8)	73.2 (11.8)	11.4	9.2
% Population ≥64 years	16.4 (4.7)	20.0 (8.1)	17.1 (3.4)	−3.6	−0.7
% Population ≤18 years	21.7 (2.5)	21.2 (3.7)	22.6 (2.2)	0.5	−0.9
% Minority	43.0 (16.2)	29.7 (13.4)	33.1 (10.9)	13.3	9.9
% College	32.3 (10.3)	25.4 (8.6)	26.6 (4.3)	6.9	5.7
% High school diploma	11.1 (3.4)	11.7 (4.7)	11.9 (2.3)	−0.6	−0.8
% Poverty	14.5 (5.3)	13.8 (5.1)	13.9 (2.8)	0.7	0.6
Crowding index	2.1 (0.7)	1.9 (1.0)	2.1 (0.5)	0.2	0
CDC Social Vulnerability Index (SVI)	58.9 (25.5)	54.8 (26.9)	55.0 (15.5)	4.1	3.9
Supplemental Security Income (SSI)	27.4 (9.7)	27.4 (9.0)	26.0 (4.6)	0	1.4
**Population chronic and health**					
% Any chronic condition	43.9 (4.9)	46.2 (4.9)	45.9 (2.8)	−2.3	−2
% Obesity	34.0 (3.5)	33.7 (4.1)	34.6 (2.6)	0.3	−0.6
% Diabetes	11.5 (1.9)	11.2 (1.7)	11.5 (1.0)	0.3	0
% Smoking	18.2 (3.0)	20.0 (2.9)	19.7 (1.4)	−1.8	−1.5
% Excessive drinking	18.2 (2.2)	19.4 (2.8)	18.6 (2.2)	−1.2	−0.4
**Health insurance**					
% No health insurance	10.8 (2.8)	11.4 (3.3)	11.1 (2.1)	−0.6	−0.3
% Public insurance	34.3 (6.4)	39.0 (9.3)	36.1 (4.0)	−4.7	−1.8
% Private insurance	67.6 (7.4)	64.9 (8.6)	66.2 (4.2)	2.7	1.4
**Google community mobility** ^b^ **(mean percentage change from baseline)**					
Workplaces	−25.3 (5.5)	−21.9 (6.1)	−22.4 (3.5)	−3.4	−2.9
Grocery and pharmacy	5.6 (10.0)	9.8 (13.9)	10.0 (9.6)	−4.2	−4.4
Residential	5.0 (1.6)	4.3 (1.4)	4.0 (1.1)	0.7	1
Retail and recreation	−3.4 (9.5)	3.0 (17.1)	3.7 (10.5)	−6.4	−7.1

The 35 treated counties had mandatory masking policies, the 69 control counties had non-mandatory policies, and the 35 synthetic controls were constructed from the control counties.

^a^Completed as at least one dose of the COVID-19 mRNA vaccines.

^b^Average percentage change in visits or time spent at specific location categories compared to a baseline period (January 3—February 6, 2020), derived from anonymized location data.

^c^The comparisons are reported as mean differences.

**Figure 2.**
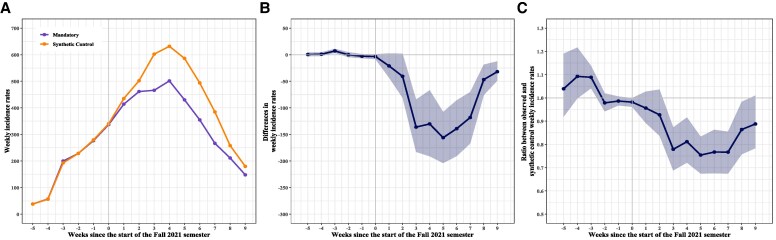
(A). Average weekly SARS-CoV-2 incidence rates per 100 000 population in counties with mandatory school masking policies and their synthetic controls during the 6 weeks before and 9 weeks after the start of the Fall 2021 semester. (B). Estimated treatment effects of the mandatory school masking policy on county-level SARS-CoV-2 incidence rates per 100,000, expressed as differences. (C). Estimated treatment effects of the mandatory school masking policy on county-level SARS-CoV-2 incidence rates (per 100 000), expressed as ratios. Note: The x-axes represent time in weeks during the study period, with zero marking the week that includes the start date of the Fall 2021 semester. Negative values indicate weeks prior to the start date (pre-intervention period), while positive values correspond to weeks following the start date (post-intervention period). The y-axes display the outcomes: in (Panel A), the county-level SARS-CoV-2 incidence rate, in (Panel B), the differences in incidence rates between counties with mandatory school masking policies and their synthetic controls, and in (Panel C), the ratio in incidence rates between counties with mandatory school masking policies and their synthetic controls. The lines show the mean values, and the shaded areas represent the 95% confidence intervals.

**Figure 3.**
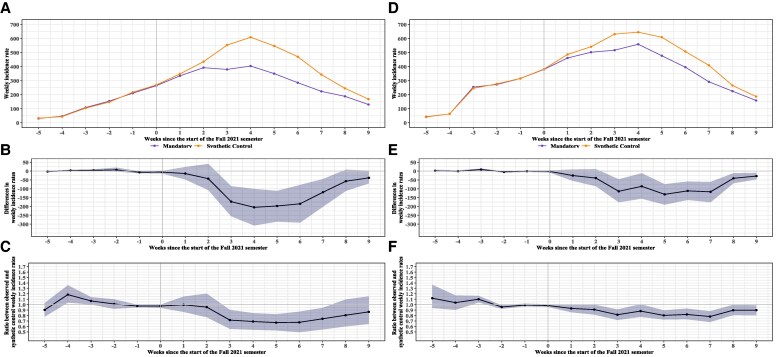
Results of stratified analysis by pre-semester SARS-CoV-2 infection levels: weekly incidence trends and estimated effects of mandatory school masking. (Panels A-C): Counties with <10% prior infection; (Panels D-F): ≥ 10%. (Panels A and D) show average weekly incidence trends per 100 000 between treated and their synthetic controls; (Panels B and E) show treatment effects on incidence per 100,000, expressed as differences; (Panels C and F) show treatment effects on incidence per 100,000, expressed as ratios.

Figure 2A illustrates the trends in average weekly SARS-CoV-2 incidence rates throughout the study period for counties with mandatory school masking policies and their synthetic controls. The nearly identical trends during the pre-intervention period indicate a close alignment between the treated counties and their synthetic controls, supporting the validity of the synthetic control construction. The 2 trends began to diverge approximately one week after the start of the Fall 2021 semester, with treated counties exhibiting lower weekly incidence rates. The largest differences were observed between the third- and sixth-weeks post-intervention, followed by a gradual convergence of the trends by week 9. Figures 2B and 3C show the estimated impact of the mandatory school masking policy on county-level weekly SARS-CoV-2 incidence rates in difference and ratio, respectively. The effect in difference ranged from 21 fewer weekly cases per 100 000 population in week one (95% CI: −45.25 to 1.67) to a peak reduction of 156 cases per 100 000 (95% CI: −205.0 to −105.5) in week 5 after the Fall 2021 semester began. After reaching its peak, the effect size gradually declined between weeks 5 and 9. By the end of the observation period, the cumulative reduction over the 9-week post-intervention period amounted to 820 fewer cases per 100 000 population (95% CI: −1184.8 to −444.0). The ratio estimates showed a consistent pattern, with the strongest effects observed between weeks 3 and 7, when case ratios ranged from 0.75 to 0.80, corresponding to approximately 20%-25% lower SARS-CoV-2 incidence associated with school mask mandates. The effect attenuated during weeks 8 and 9, with point estimates remaining below 0.9 but confidence intervals crossing 1.0 by week 9. Over the study period, the relative cumulative reduction in incidence was 9.4% (95% CI: 7.3%-11.8%).

Similar trends in outcomes and intervention effects were observed across subgroups, with stronger effects observed in counties with lower prior population SARS-CoV-2 infection percentages, lower population density, and higher community mobility during the 6 weeks before the start of the Fall 2021 semester. The most pronounced effects were observed in counties with prior population SARS-CoV-2 infection percentages below 10%, where the largest weekly reduction occurred at week 4, with an estimated decrease of approximately 200 fewer weekly cases per 100 000 population (95% CI: −310.8 to −100.5). The cumulative reduction over the 9-week post-intervention period reached 1031 fewer cases per 100 000 population (95% CI: −1668.5 to −353.6), corresponding to a relative reduction of 9.9% (95% CI: 6.5%—13.6%) (Figure 3A–3C). In contrast, counties with higher population SARS-CoV-2 infection percentages before the intervention experienced a less pronounced effect, with a cumulative reduction of 695 cases per 100 000 population, with a relative reduction of 9.2% (95% CI: 6.5%—12.0%) over the same period (Figure 3D and 3E). Results from analyses stratified by population density and community mobility are presented in Figures S4 and S5. Results of placebo check and sensitivity analyses assessing the robustness of the results are presented in Supplementary Materials (Figures S6-S11).

## Discussion

Existing evidence on school masking mandates is substantial but heterogeneous. For example, a large Massachusetts study observed increases in student and staff infections after school districts lifted mask mandates in early 2022.^2^ A US systematic review found low to moderate certainty evidence regarding student mask mandates and transmission reduction.^22^ Other work has found lower seroconversion rates among children in districts with mask requirements.^23^ However, relatively few studies estimate the broader community spill-over effects of school masking, or utilize quasi-experiments with county-level controls. Our study addresses this gap by implementing a synthetic-control design across counties, targeting communitywide incidence rather than school-only outcomes. Our analysis focused on the fall 2021 school reopening, a period when most broad community restrictions (eg, business closures, gathering bans, strict quarantine policies) had been lifted or inconsistently applied, leaving school mask mandates as one of the most consistently implemented interventions. This distinct policy context makes the period well-suited for examining the short-run effects of school masking. We acknowledge that the subsequent Omicron surge was substantially larger in magnitude, and our findings should not be extrapolated to later phases of the pandemic with different variants or higher population immunity. Nonetheless, evaluating this reopening window provides policy-relevant evidence on the role of school masking in reducing community transmission when schools resume in-person instruction under conditions where other mitigation measures are largely absent. In doing so, we find that school-masking policies are associated with reduced community transmission, thereby adding evidence that the benefits of school masking extend beyond the classroom and contribute to reducing broader community transmission. This complements prior work by demonstrating potential spillover effects to broader populations outside the school environment.

Our subgroup analyses revealed that the effectiveness of school masking policies varied based on community characteristics. The intervention effect was most pronounced in counties with lower pre-intervention SARS-CoV-2 infection percentages, where the cumulative reduction over 9 weeks reached 1031 cases per 100 000 population. This suggests that counties with larger immunological naïve population may benefit more from school masking policies, possibly due to reduced baseline community spread and greater capacity to mitigate surges. Additionally, the intervention effect was greater in counties with lower population density where K-12 schools may represent a larger proportion of total community interactions and in counties with higher community mobility where reducing school transmission may have a broader impact due to frequent movement in the community. These findings highlight the importance of contextual factors in shaping the effectiveness of NPIs and align with prior research suggesting that demographic, social, and behavioral characteristics can modify the impact of public health interventions.

The observed reduction in community incidence rates associated with mandatory school masking policies supports the inclusion of such interventions in broader strategies to control infectious disease transmission. However, that finding alone does not impart judgement on whether required masking policies were a necessity at the time. Policymakers should consider the context-specific effectiveness of school masking policies, particularly in areas with lower pre-existing transmission or other favorable community characteristics. Furthermore, the diminishing effect observed after week 5 should be interpreted with caution. The ratio estimates remained relatively stable through weeks 6 and 7, even as overall incidence declined, suggesting that the attenuation in case difference may largely reflect the natural downturn in community transmission rather than a waning effect of masking. However, as incidence dropped further toward the end of the study, the ratio effect approached toward 1.0. Although our analysis cannot fully disentangle these mechanisms, the findings suggest that masking was most impactful during periods of higher transmission. To sustain low transmission over time, complementary strategies such as vaccination campaigns and expanded testing remain important components of a comprehensive public health response.

A key strength of this study is the use of the SCM, which mitigates unmeasured confounding by creating well-matched synthetic controls for treated counties. The strong pre-intervention alignment between treated and synthetic control counties supports the validity of our findings, and results were robust across sensitivity analyses using alternative donor specifications and lag structures. The inclusion of a broad range of county-level covariates and stratified subgroup analyses provides a nuanced understanding of how community characteristics influence the effectiveness of school masking policies. PCA further stabilized synthetic control construction and reduce overfitting in a small-sample setting. We emphasized outcome alignment as the primary diagnostic of match quality and used PCA solely to stabilize the covariate space. Nonetheless, synthetic control estimates can be sensitive to epidemic variability. Even with good pre-intervention fit and robust placebo checks, some post-intervention divergence may reflect stochastic disease dynamics rather than policy effects. Accordingly, our estimates should be interpreted as evidence of association within this specific policy and epidemiologic context.

This study has several limitations. First, the overrepresentation of counties from southern U.S. regions in our final sample may limit the transportability of our findings to other regions with different sociodemographic characteristics or policy environments. Second, we restricted donor pools to the same or adjacent states to enhance plausibility, recognizing that proximity does not guarantee demographic or policy similarity. This regional constraint improved interpretability, while match quality was ensured through covariate and outcome alignment. Third, the reliance on publicly available data may introduce misclassification bias in masking policies, although our validation process showed high consistency in exposure classification. Fourth, while SCM minimizes confounding, unmeasured factors specific to treated counties may still influence the results. Finally, we could not stratify incidence by age or adjust for other COVID-19 policies due to data unavailability. Our outcome therefore reflects total community incidence, and the estimates may capture the combined effects of school masking and any concurrent measures.

Future studies should explore the long-term effects, including possible negative impact on learning, of school masking policies and their interaction with other NPIs, such as vaccination and testing strategies. Additionally, investigations into the differential impact of masking policies on specific subpopulations, such as students, teachers, and families, could provide insights into more targeted policy development.

## Supplementary Material

qxaf233_Supplementary_Data
